# Control of cell colony growth by contact inhibition

**DOI:** 10.1038/s41598-020-62913-z

**Published:** 2020-04-21

**Authors:** Simon K. Schnyder, John J. Molina, Ryoichi Yamamoto

**Affiliations:** 1 Fukui Institute for Fundamental Chemistry, Kyoto University, Kyoto, 606-8103 Japan; 2 Department of Chemical Engineering, Kyoto University, Kyoto, 615-8510 Japan; 3 Institute of Industrial Science, The University of Tokyo, Tokyo, 153-8505 Japan

**Keywords:** Computational biophysics, Motility, Biological physics, Cell division, Cell polarity, Cell migration, Collective cell migration, Epithelial-mesenchymal transition

## Abstract

Contact inhibition is a cell property that limits the migration and proliferation of cells in crowded environments. Here we investigate the growth dynamics of a cell colony composed of migrating and proliferating cells on a substrate using a minimal model that incorporates the mechanisms of contact inhibition of locomotion and proliferation. We find two distinct regimes. At early times, when contact inhibition is weak, the colony grows exponentially in time, fully characterised by the proliferation rate. At long times, the colony boundary moves at a constant speed, determined only by the migration speed of a single cell and independent of the proliferation rate. Further, the model demonstrates how cell-cell alignment speeds up colony growth. Our model illuminates how simple local mechanical interactions give rise to contact inhibition, and from this, how cell colony growth is self-organised and controlled on a local level.

## Introduction

Cells move collectively and proliferate^1–6^ as the embryo develops during morphogenesis^7^, as cancer spreads or as wounds close^8–10^. The way in which migration and proliferation interact with each other is complex^11^. Essential for the regulation of these processes is contact inhibition of locomotion (CIL), which describes the tendency of cells to stop migration or change direction when coming into contact with other cells^12–16^. CIL has been shown to enable cells to collectively migrate^14^, follow chemical gradients^17,18^, and disperse. It is now believed that CIL helps the  control of collective tissue migration^16,17,19,20^, tissue growth^21,22^, morphogenesis^14^, wound healing and tumour development^23^.

Potentially distinct from CIL is contact inhibition of proliferation (CIP) which refers to the suppression of cell divisions in dense regions of tissues^24–26^, which in turn regulates their growth. There is evidence that CIP does not require direct cell contact^27–30^ and as a consequence the effect is also called “density dependent inhibition of cell growth”^31^.

Modelling approaches for cell migration are manifold^32^, ranging from strongly idealised models for single cells crawling on substrates^33–35^ to cells in confluent tissues^36^ to continuum theories^37–39^. The collective behaviour of cells is under intense study and many questions about contact inhibition are still open^16,26^.

In order to reduce the complexity of the systems under study, it is valuable to investigate well controlled model systems. One such model system deals with the crawling and proliferation of a monolayer of cells seeded onto a substrate. How a few cells develop into an extended colony has been investigated in a recent experiment^21^, as well as in simulations, e.g.^22,40–48^. The colony in said experiment consists of of Madin-Darby canine kidney epithelial (MDCK) cells^49^ and exhibits two growth regimes. In the beginning the colony’s area grows exponentially with time, followed by sub-exponential growth. Similar growth stages are also found in tumour growth dynamics, e.g.^50,51^ In the former regime, cells are highly mobile and divide frequently, while in the latter, both the motion and proliferation of the cells becomes suppressed, linking the transition to contact inhibition. Such a transition is readily found in simulations if the proliferation rate of cells is locally coupled to density or available space^40,41,44^, or stress^37^.

Previously, we developed a minimal, mechanical model for crawling cells in which contact inhibition of locomotion arises from the internal mechanics of the cells^52^. Our model focuses purely on the cell mechanics, since mechanical forces inside of and between cells are now understood to be of crucial importance for the understanding of cell dynamics^9,21,53–58^. Our model differs from previous models in that it incorporates a coupling of motility to cell polarisation, and asymmetric cell shapes into a minimal model; both of which influence mechanical interactions between cells. We found that the model naturally exhibits a range of realistic behaviours, including the emergence of collective migration, without including an explicit alignment term. Our model is thus a natural candidate to investigate colony growth.

In this paper, we extend our model to include cell proliferation in such a way that the motility and division dynamics are entirely governed by the internal dynamics of the cells. The cells cycle between a motile phase and a division state. In the division state, the cells attempt to proliferate by making space for two cells; otherwise, the cell division is aborted. This naturally gives rise to a form of contact inhibition of proliferation. Similar approaches have been used e.g. by^40,44^.

We find that our model reproduces the typical colony dynamics; with exponential growth at short times turning into sub-exponential growth with a constant boundary speed at long times. Coinciding with this transition, the average cell speed decreases strongly, because of CIL occurring in the inside of the colony. As a result of contact inhibition, cells close to the boundary have higher speeds and proliferation rates. By varying the proliferation rate over a wide range, we then identify simple scaling relations for both regimes and the crossover between them. We had previously found that cell shape has a strong effect on cell collisions and that cells with large fronts align and coherently migrate^52^. In this work, we now demonstrate that cell with large fronts orient themselves away from the colony, which enhances the speed of colony expansion.

## Model

**Figure 1:**
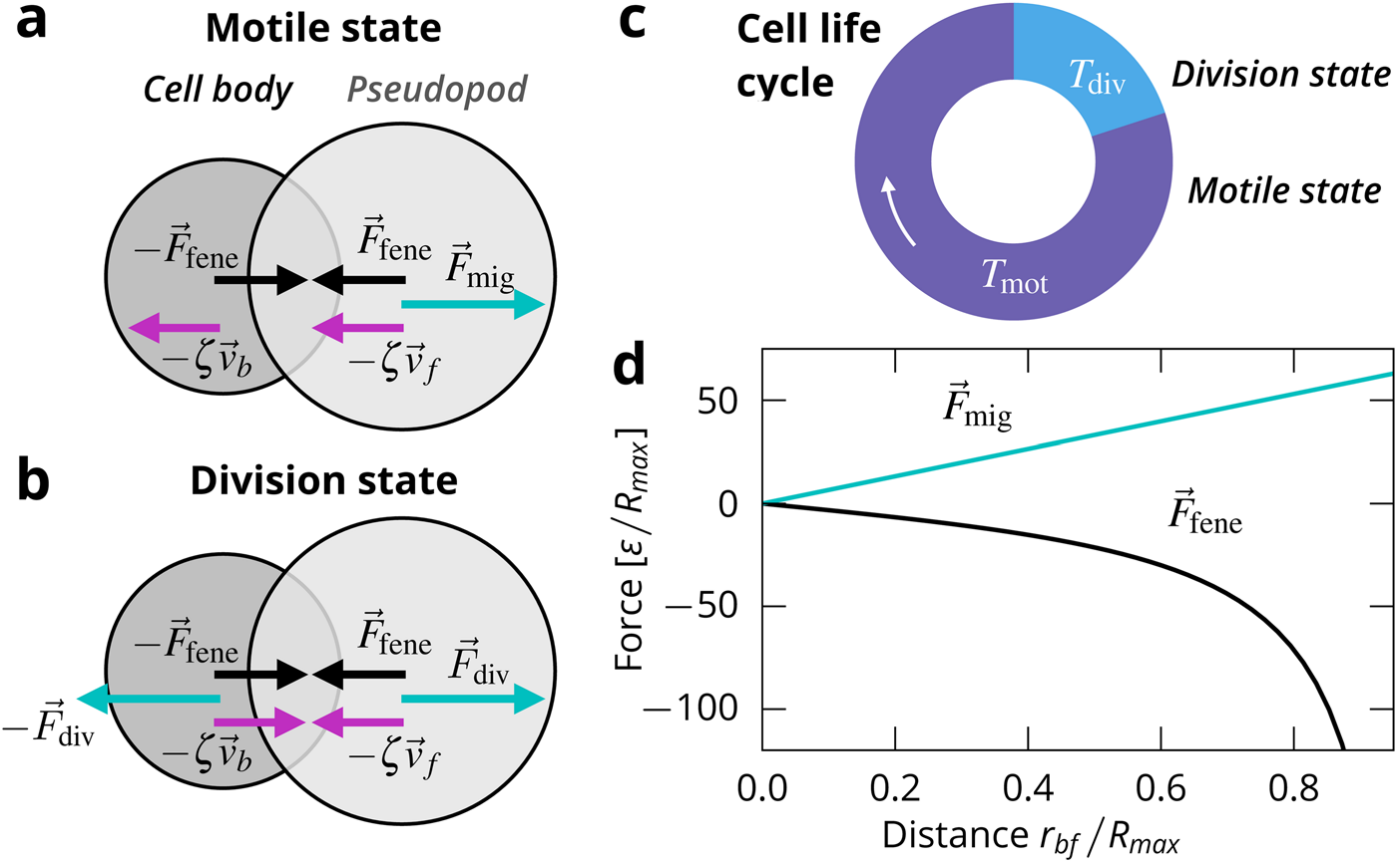
Model overview. (**a**) Schematic of the cell model in the motile state. (**b**) Schematic of the cell model in the division state (**c**) Illustration of the cell life cycle. (**d**) Forces acting on the disks being separated a distance ${r}_{bf}=|{\overrightarrow{r}}_{bf}|$.

We build on a model for crawling cells^52^, and include cell division. Each cell consists of two disks with distinct roles, see Fig. 1. One models a pseudopod and is at the front of the cell (index $f$), the other disk represents the cell body and is at the back of the cell (index $b$). The positions of the disks ${r}_{f}$ and ${r}_{b}$ define the distance between the elements and the orientation of the cell ${\overrightarrow{r}}_{bf}={\overrightarrow{r}}_{f}-{\overrightarrow{r}}_{b}$.

The dynamics of the cells are coarse-grained over the typical idealised crawling cycle^59^. The substrate exerts a drag force $-{\zeta }_{i}{\overrightarrow{v}}_{i}$ on the disks, with ${\overrightarrow{v}}_{i}$ being the velocity of the disks ($i\in f,b$) and ${\zeta }_{i}$ being the respective drag coefficients. For simplicity, we set ${\zeta }_{1}$ = ${\zeta }_{2}$ = $\zeta$. We employ the commonly made assumption that the friction with the substrate is the dominant contribution to friction in the system, e.g. by^60^, and neglect intracellular friction and friction between different cells.

It is now understood that the shape of cells can be highly variable and has a strong influence on their collective behaviour^36,61–66^. In our model, the cell shape is determined by the configuration of the two disks with diameters ${\sigma }_{f}$ and ${\sigma }_{b}$, and can be understood as representing a statistical average over the real cell shape. Such a coarse graining of the highly variable shape of real cells is a promising approach for cells whose shape does not deviate too much from the average, e.g. for epithelial cells. In our previous work, we showed that the present model exhibits an alignment transition in coherent migration as a function of cell shape^52^. Cells with a small front disk (as compared to the back disk) are bad at moving apart after colliding, while cells with a large front disk push each other out of their path, which over time leads to global alignment. In this sense, cells with a large front disk in our model exhibit another form of contact inhibition. For this work, we mainly investigate cells with a shape ratio of ${\sigma }_{b}/{\sigma }_{f}=0.79$ which exhibits realistic alignment and migration behaviour^52^.

In our previous work, interactions between cells were purely repulsive. Here, we introduce new, adhesive interactions between cells. The adhesiveness of the potential is characterised by its well depth ${\varepsilon }_{{\rm{well}}}$ in respect to the potential height ${\varepsilon }_{{\rm{core}}}$. The force acting on a disk *α* by other cells is denoted ${F}_{cc,\alpha }$ ($\alpha \in [b,f]$). For details, see Methods.

### Cell life cycle

Each cell switches independently between two states, a motile state and a division state. The duration of the motile state is determined when switching to it from the division state, by drawing a random number from a normal distribution with mean ${T}_{{\rm{mot}}}$ and standard deviation ${T}_{{\rm{mot}}}$/2. With this distribution we model natural fluctuations in cell cycle duration. The duration of the division state is held constant at ${T}_{{\rm{div}}}$. On average, the duration of the whole cell cycle is thus $T={T}_{{\rm{div}}}+{T}_{{\rm{mot}}}$.

In the motile state, the cell behaves as in ref. ^52^. The front disk exerts a migration force ${F}_{{\rm{mig}}}$ in the direction of the orientation of the particle, while the back disk is passive. The migration force is given by ${\overrightarrow{F}}_{{\rm{mig}}}({\overrightarrow{r}}_{bf})=m{\overrightarrow{r}}_{bf}$ with motility strength $m$, see Fig. 1(a,d). The connection between the disks is modelled as a finitely extensible nonlinear elastic (FENE) spring^67^, which gives rise to a contracting force between the disks ${\overrightarrow{F}}_{{\rm{fene}}}({\overrightarrow{r}}_{bf})=-\,\kappa {\overrightarrow{r}}_{bf}\mathrm{/[1}-{({r}_{bf}/{R}_{{\rm{\max }}})}^{2}]$  with coupling parameter $\kappa$ determining the strength of the contraction, and ${R}_{{\rm{\max }}}$ the maximum distance between the two disks.

In the division state, cells attempt to make space for two daughter cells. Cells only divide if at the end of the division state the cell extension reaches a division threshold ${R}_{{\rm{div}}}$. Coupling cell proliferation to cell area is an idealisation of the observation that larger cells divide more frequently in experiments^21,68^ and models contact inhibition of proliferation.

Cells elongate by having both disks enact the same migration force ${F}_{{\rm{div}}}$ with opposite sign. For convenience we set ${F}_{{\rm{div}}}={F}_{{\rm{mig}}}$. The contracting force between the disks remains unchanged. Cells do not migrate in the division state. To make space for the new cells, we increase the size of the smaller of the two disks linearly until it matches the size of the larger disk at the end of the state. After a successful division we construct two cells in place of the original cell’s disks. We randomly displace the new cell’s disks to randomise the orientation of the new cells. If ${r}_{bf} < {R}_{{\rm{div}}}$ at the end of the division state, the cell division is aborted, the cell contracts again, and the migration state is entered.

### Equations of motion

For each of the cells, we now have two coupled non-linear equations of motion, assuming overdamped dynamics

1 $$\begin{array}{rcl}\frac{{\rm{d}}}{{\rm{d}}t}{\overrightarrow{r}}_{b} & = & \frac{1}{\zeta }(-\,{\overrightarrow{F}}_{{\rm{fene}}}({\overrightarrow{r}}_{bf})-\chi {\overrightarrow{F}}_{{\rm{mig}}}({\overrightarrow{r}}_{bf})+\sum _{{\rm{neigh}}.}{\overrightarrow{F}}_{{\rm{cc}},{\rm{b}}}),\\ \frac{{\rm{d}}}{{\rm{d}}t}{\overrightarrow{r}}_{f} & = & \frac{1}{\zeta }({\overrightarrow{F}}_{{\rm{fene}}}({\overrightarrow{r}}_{bf})+{\overrightarrow{F}}_{{\rm{mig}}}({\overrightarrow{r}}_{bf})+\sum _{{\rm{neigh}}.}{\overrightarrow{F}}_{{\rm{cc}},{\rm{f}}}),\end{array}$$

with $\chi =1$ in the division state and $\chi =0$ in the motile state.

Apart from the randomised positions of daughter cells’ disks, our model does not include random forces. This is a reasonable assumption when collisions (and cell division) dominate the dynamics^69,70^. In the migration state, the cell is only motile when its disks have some separation, ${r}_{bf} > 0$, and thus when its shape deviates from a circle. This kind of coupling of motility and deformation is typical in migratory cells^71^.

If the cell is in the motile state for long enough, it then enters a steady state with constant extension ${r}_{bf}^{{\rm{ss}}}$ and constant speed ${v}^{{\rm{ss}}}$ in which the forces acting on the cell balance^52^.

## Results

### Colony growth

**Figure 2:**
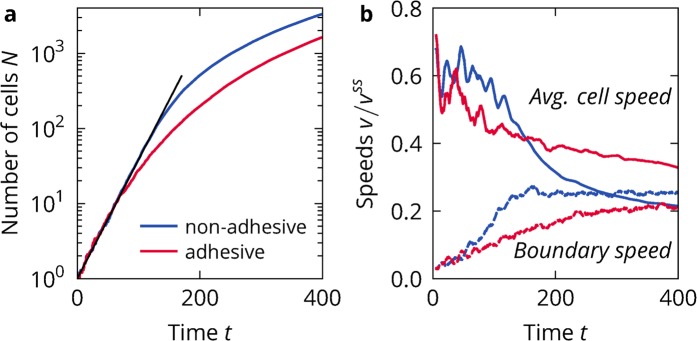
Colony growth for non-adhesive and adhesive cells. (**a**) Size of the colony in number of particles $n$ against time $T$ for ${T}_{{\rm{mot}}}=16$ and ${T}_{{\rm{div}}}=3$. The black line marks exponential growth, assuming that all cell division attempts are successful, see Eq. (2). (**b**) Speed of the colony boundary and average cell speed of the same simulations.

At first, we simulated cell colonies of non-adhesive cells to determine what results the simplest possible interactions yield in our model, see Fig. 2a) and Video S1 (Supplementary Materials). At early times, the colony grows exponentially, but eventually crosses over into sub-exponential growth. In the exponential regime, all cell division attempts are successful. Since cells attempt to double with a rate of $T={T}_{{\rm{mig}}}+{T}_{{\rm{div}}}$ and we always start with one cell at $t=0$, the number of cells grows as

2 $$N(t)={2}^{t/T}=\exp \left(\frac{\mathrm{ln}\,2}{T}t\right).$$

In the experiment^21^, the sub-exponential growth is characterised by the boundary of the colony moving outwards at a constant speed. If $R(t)$ is the radius of the approximately circular colony, then the outwards speed of the boundary can be extracted from the area $A(t)=\pi R{(t)}^{2}$ of the colony as

3 $${v}_{B}=\frac{{\rm{d}}R(t)}{{\rm{d}}t}=\frac{{\rm{d}}}{{\rm{d}}t}\sqrt{A(t)/\pi }\mathrm{}.$$

The area of the colony can be calculated from the areas of all the individual cells, for which we use Eq. (11). At long times, the speed of the boundary saturates in our simulation to a constant speed as well, see Fig. 2b). We find the speed to be ${v}_{B}\approx 0.2{v}^{{\rm{ss}}}$. The speed of cells allows quantifying the activity of the colony over time. In the exponential regime, the average cell speed is constant and then in the sub-exponential regime decreases over time, eventually dropping below the boundary speed, see Fig. 2b). The transition in the average cell speed occurs at the same time as the transition of the boundary speed. All of this is qualitatively similar to what is observed in the experiment.

Adhesive cells exhibit similar growth dynamics, see Fig. 2 and Video S3 (Supplementary Materials). The slope of the exponential regime is the same, with all divisions being successful at early times. The average cell speed in the exponential regime remains unchanged as well. However, the exponential regime only extends until the colony consists of tens of cells. The transition to a constant boundary speed takes much longer, as does the slowing of the average cell speed. On average, we find cells to be faster, but the colony to expand slower.

### Radial analysis

**Figure 3:**
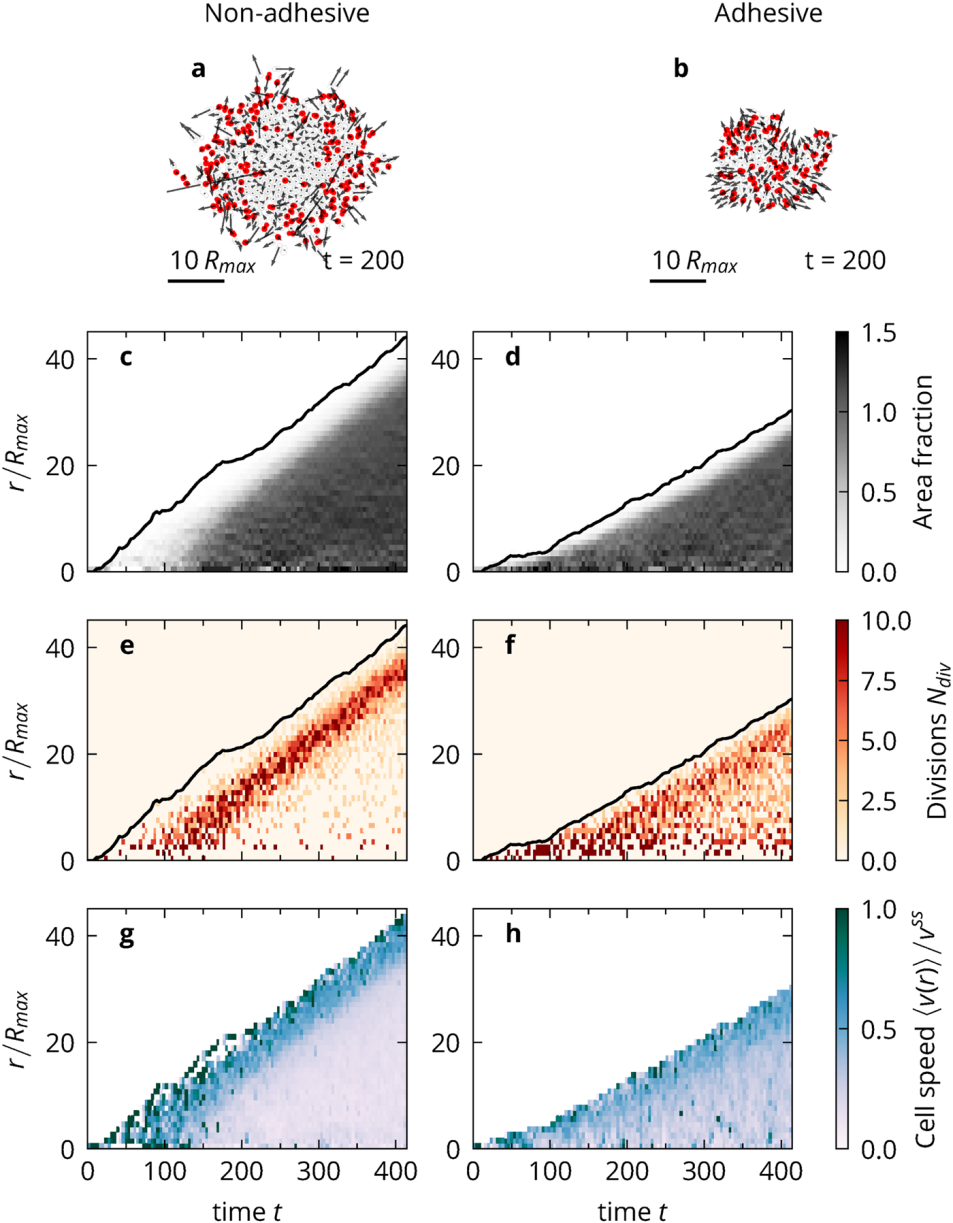
Radial analysis. (**a**) Simulation snapshot of a colony of non-adhesive cells. Cells marked in red were created via cell proliferation in the preceding time span Δ*t* = 20. Cell speeds are given as arrows. (**b**) The same as (**a**) but for adhesive cells. (**c**) Cell density for the non-adhesive cell simulation as a function of distance to the colony centre and time. The distance of the outermost cell for each point in time is indicated by the black line. (**d**) The same as (**c**) but for adhesive cells. (**e**) Number of cell divisions per unit time and unit area for the non-adhesive cell simulation as a function of distance to the colony centre and time. (**f**) The same as (**e**) but for adhesive cells (**g**) Average cell speed for the non-adhesive cell simulation as a function of the distance from the colony centre and time. (**h**) The same as (**g**) but for adhesive cells. Figure created with Matplotlib v.3.1.0 (https://matplotlib.org/)^75^.

To understand the growth of the colonies in more detail, we look at the spatial distribution of the following key quantities: cell density, cell divisions, and cell speed. For this we analyse one exemplary simulation with non-adhesive and adhesive cells. Non-adhesive cells form colonies with a diffuse boundary, with some cells even escaping, Fig. 3a), whereas adhesive cells form a denser colony, with no cells escaping the bulk, see Fig. 3b).

For the non-adhesive cells, we find that in the exponential regime ($t < 150$) the density is quite low but later quickly reaches a high value in the bulk of the colony, Fig. 3c). The boundary of the colony remains diffuse with a low density and constant width over the whole simulation. The colony of adhesive cells is different at early times, see Fig. 3d), because it is dense and cohesive from the beginning. At long times, the adhesive colony appears qualitatively quite similar to the non-adhesive colony, with the same bulk density, albeit with a sharper boundary.

We then calculated the radial distribution of successful cell divisions ${N}_{{\rm{div}}}(r,t)$, see Methods. In the exponential regime, all attempted divisions are successful, and thus are distributed homogeneously over the colony, Fig. 3e,f). For non-adhesive cells, divisions at later times mostly occur in a ring of constant thickness at the boundary of the colony, where there is more space available to the cells, see Fig. 3a,e). This is a result of our cell division mechanism which naturally gives rise to contact inhibition of proliferation. Cells in the bulk cannot make the necessary space for the cell division to occur, except for the colony centre, where space becomes available as cells migrate outwards. For adhesive cells we find a similar pattern, see Fig. 3f), where a ring of increased proliferation probability can still be discerned. However, the probability of a division occurring at the boundary is considerably reduced, because the local density tends to be higher as compared to the non-adhesive colony. In addition, cell divisions are more frequent in the bulk of the colony. The reason for this becomes clearer with an analysis of the cell speeds.

Non-adhesive cells are mostly mobile at the boundary of the colony, with motion being strongly suppressed by CIL in the colony bulk, see Fig. 3a). The cells on the boundary are on average pointing away from the colony centre, but there is considerable local variation due to contact inhibition of locomotion after collisions and noise introduced by cell divisions. Cells can momentarily obtain high speeds after a successful cell division when they move away from the other daughter cell.

In the exponential regime, all cells are mobile, but in the sub-exponential regime, only cells at the boundary exhibit speeds close to the single-cell steady state speed ${v}^{{\rm{ss}}}$, Fig. 3g). Motion in the bulk is suppressed strongly by contact inhibition of locomotion. In comparison, adhesive cells are slower on the border but more mobile in the bulk and more aligned with each other, see Fig. 3b,h). We attribute this to the cells at the boundary being held back by the cells at their back and in turn the cells of the bulk being pulled outwards by the boundary cells. This is commonly called the “tug of war” between cells^9,57,72^. The tug of war leads to a different bulk structure between the colonies, even though the densities are similar, Fig. 3a,b). There is more free space in the non-adhesive colony and cells are more compressed due to contact inhibition of locomotion. The adhesive colony, on the other hand, is fully cohesive, with cells always being at contact and pulling on each other and therefore, on average, more extended. This is reflected in the higher cell speeds in the bulk of the adhesive colony. As a result, it becomes easier for the adhesive cells in the bulk to reach the division threshold. In conclusion, we find that cell-cell adhesion considerably alters the colony structure on a local level, while leaving the qualitative colony dynamics with the two growth regimes unchanged.

### Variation in the cell cycle

**Figure 4:**
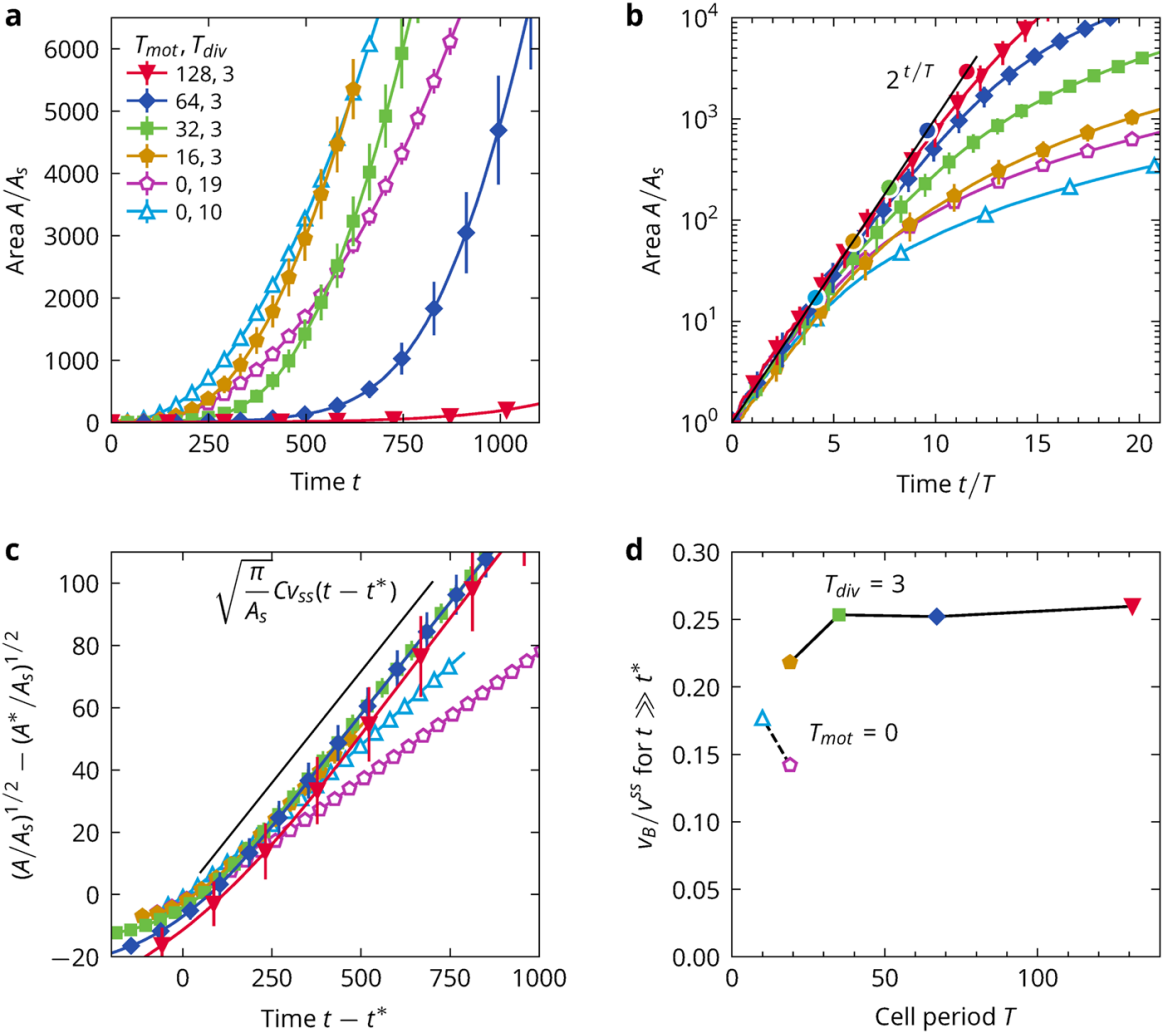
Variation in the cell cycle. (**a**) Area of the colony for a range of ${T}_{{\rm{mot}}}$ against time. Error bars calculated from the standard deviation of colony sizes for the independent simulation runs are marked as vertical lines at regular intervals. (**b**) Area of the colony for a range of ${T}_{{\rm{mot}}}$ against rescaled time. The black line marks the exponential growth expected for when all cell division attempts are successful, Eq. (4). The coloured circles mark the estimates for the crossover time ${T}^{\ast }$ and crossover colony size ${A}^{\ast }$, as determined from Eqs. (6) and (8). The simulations with ${T}_{{\rm{mot}}},{T}_{{\rm{div}}}=16,3$ and 0, 19 share the same crossover. (**c**) Scaling plot to expose linear long-time growth. The black line gives the asymptote of Eq. (10). (**d**) Boundary speed against the cell cycle period $T$.

We illustrate in the following that at short times, the colony dynamics is entirely determined by the proliferation rate, whereas at long times, the dynamics are determined by the migration properties of the cells. For this purpose we vary the durations of the migration and division states of the cells, Fig. 4a). For comparison, we also simulated the situation where the cells are not migrating at all, but divide regularly.

Since the colony area grows as

4 $$A(t)\approx {A}_{s}N(t)={A}_{s}{2}^{t/T}$$

at early times, we find that all simulations collapse onto one asymptote when time is rescaled by $T={T}_{{\rm{mot}}}+{T}_{{\rm{div}}}$, see Fig. 4b). Therefore, while contact inhibition of proliferation is weak, the dynamics are purely determined by the rate of cell divisions 1/*T*. As a consequence, it also does not play a role whether the cells are migratory or not.

However, this simple rescaling cannot collapse the data at long times, as the time at which growth becomes sub-exponential varies between the simulations. To calculate those crossover times, we make use of the observation that the boundary speed of the colonies is limited. In the exponential regime, the boundary speed increases as, see Eqs. (3) and (4),

5 $${v}_{B}(t)=\frac{{\rm{d}}}{{\rm{d}}t}\sqrt{\frac{A(t)}{\pi }}\approx \frac{\mathrm{ln}\,2}{2T}\sqrt{\frac{{A}_{s}}{\pi }}{2}^{t\mathrm{/2}T}\mathrm{}.$$

The crossover time ${t}^{\ast }$ is reached when the colony reaches the terminal boundary velocity which we assume to be proportional to the steady state speed of a single cells, ${v}_{B}^{\ast }=C{v}^{{\rm{ss}}}$ with some factor $C$. Then we obtain

6 $${t}^{\ast }=T\,{\log }_{2}\left[\frac{4\pi }{{(\mathrm{ln}\mathrm{2)}}^{2}}\frac{{(C{v}^{{\rm{ss}}}T)}^{2}}{{A}_{s}}\right]$$

From the data, we find that $C=0.25$ holds generally for migrating cells, see Fig. 4d). With this value for $C$, the crossover times correctly mark the transitions to sub-exponential growth for all simulations, see Fig. 4b). From Eq. (6), we see that the crossover is most strongly influenced by the duration of the cell cycle, followed by the crawling speed of the cells. At the crossover, the colony is of size ${A}^{\ast }={A}_{s}{2}^{{t}^{\ast }/T}$. This is equivalent to

7 $${t}^{\ast }=T\,{\log }_{2}({A}^{\ast }/{A}_{s}).$$

and thus

8 $${A}^{\ast }=\frac{4\pi }{{(\mathrm{ln}\mathrm{2)}}^{2}}{(C{v}^{{\rm{ss}}}T)}^{2}\,{\rm{and}}\,{R}^{\ast }=\frac{2}{\mathrm{ln}\,2}C{v}^{{\rm{ss}}}T$$

At long times, $t\gg {t}^{\ast }$, the radius of the colony grows with constant speed $C{v}^{{\rm{ss}}}$, so we find that approximately it must hold that

9 $$R(t)\approx {R}^{\ast }+C{v}^{{\rm{ss}}}(t-{t}^{\ast }\mathrm{)}.$$

Rewritten for the colony area, we have

10 $$\sqrt{A(t)}-\sqrt{{A}^{\ast }}=\sqrt{\pi }C{v}^{{\rm{ss}}}(t-{t}^{\ast })$$

This long-time scaling is exposed in Fig. 4c). Most of the data collapse onto a master function that is close to Eq. (10). The two simulations that deviate from this are the two cases in which the cells do not actively migrate and thus necessarily violate the scaling.

We therefore find that if the cells are actively migrating, the colony boundary moves at a constant speed determined by cell motility, and that if the cells are not migrating, long time growth becomes severely suppressed. Or stated differently, regardless of the proliferation rate, at long times the colony expands as fast as the cells on the boundary are able to migrate away from the colony centre.

Notably, the boundary speed is smaller than the steady state speed of a solitary cell ${v}^{{\rm{ss}}}$ by the factor C. This has two reasons: (1) the cells on the boundary adhere to the rest of the colony and are pulled back by them (2) The cells are not perfect at orienting themselves away from the colony.

Match to the experiment: MDCK cells require about one hour to travel their own length, with typical speeds being $v\sim 10\,\mu m$ and cells being of size $s\sim 10\,\mu m$ and they divide roughly every ${\tau }_{2}\sim 18\,h$^21^. This yields a ratio between the two time scales of $v{\tau }_{2}/s\approx 18$. For the model cells, the same ratio is given by ${v}^{{\rm{ss}}}T/{R}_{{\rm{\max }}}$ which evaluates to $16$ for ${T}_{{\rm{mot}}}=64$. This parameter choice therefore presents our best match to the experiment. For ${T}_{{\rm{mot}}}=64$, we find that the crossover is reached at a colony size of ~1000 cells after ≈10 full cell cycles, see Fig. 4b). This compares well with the 5–6 days, i.e. ≈7 full cell cycles, that the cells undergo in the experiment until the crossover occurs at a similar colony size, considering that our colonies originate from a single cell while the experiment starts with multiple cells.

### Influence of cell shape

**Figure 5:**
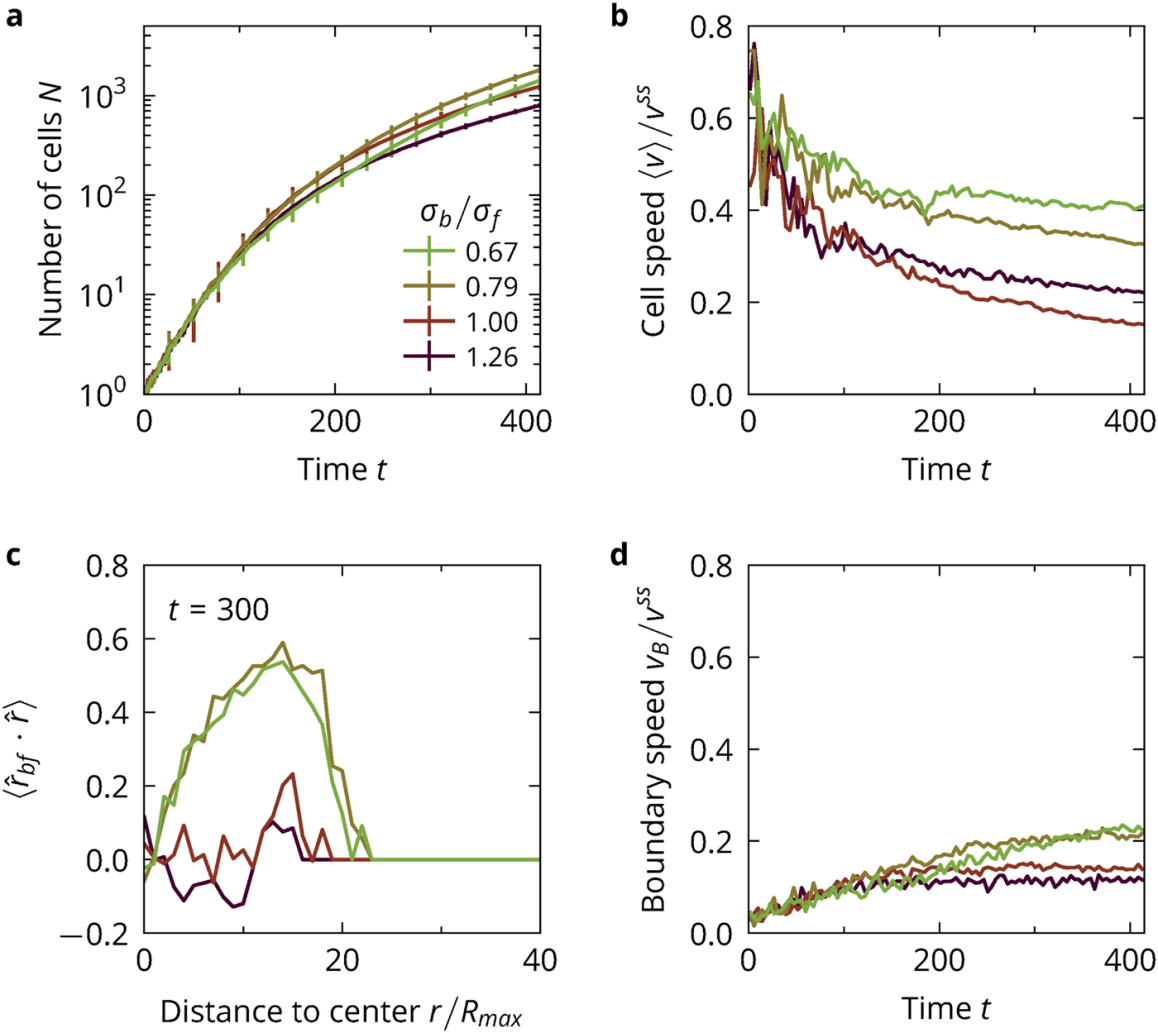
Colony growth for different cell shapes. (**a**) Number of cells as a function of time with error bars given by the standard deviation between independent simulation runs. (**b**) Average cell speed over time. (**c**) Average orientation of cells in respect to the direction pointing directly away from the colony center at time $t=300$. Positive values indicate cells pointing away from the colony, negative values indicate cells pointing towards the colony. Larger values mean that cells are better aligned and/or more extended. (**d**) Average speed of the colony boundary.

We investigate the interplay of the alignment transition with colony growth, see Fig. 5. The exponential growth regime is found to be independent of shape, see Fig. 5a), while at long times colonies with large-front cells tend to expand more rapidly. Colonies for ${\sigma }_{b}/{\sigma }_{f}=0.67$ are about 3 times larger than for ${\sigma }_{b}/{\sigma }_{f}=1.26$ at the end of a simulation. For all times, cells with larger fronts tend to be faster, see Fig. 5b). This is because cells with larger fronts are better at aligning away from the colony, as the average orientation of the cells in respect to the colony $\langle {\hat{r}}_{bf}\cdot \hat{r}\rangle$ shows, Fig. 5c). Cells with small fronts tend to be slightly aligned towards the colony centre. If the cells have large fronts, they tend to be aligned away from the colony, with alignment getting more pronounced towards the boundary of the colony. In the growth of a colony, the alignment of the cells due to shape acts as a mechanism for orienting the cells away from the colony, which is what is expected from cells exhibiting contact inhibition of locomotion^16^. As a result, the colonies of cells with large fronts expand much more quickly in the sub-exponential regime, see Fig. 5d) with the boundary speed being about twice as large for ${\sigma }_{b}/{\sigma }_{f}=0.67$ than for ${\sigma }_{b}/{\sigma }_{f}=1.26$. The described effects are more pronounced for non-adhesive cells, see Fig. S2 (Supplementary Materials).

### Neighbour information

**Figure 6:**
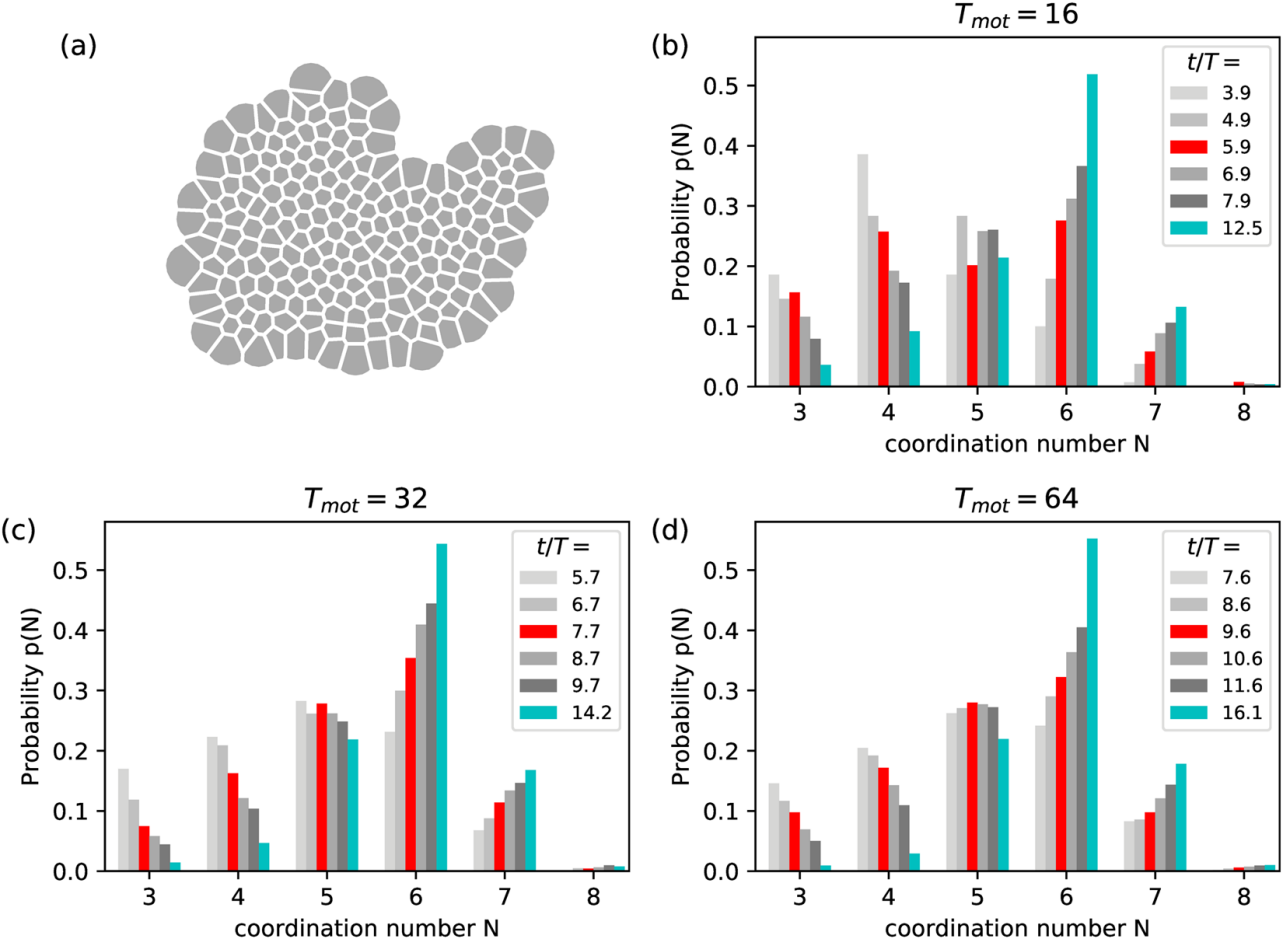
Voronoi analysis. (**a**) Voronoi tessellation of a simulation snapshot of a colony of adhesive cells with ${T}_{mot}=16$ at $t=200$. The voronoi cells are truncated at the maximum interaction radius of the cells, see text. (**b**–**d**) Histogram of the coordination number of the cells as derived from the Voronoi tessellation for a range of times, chosen relative to ${T}^{\ast }$. The shown times approximately obey $(t-{T}^{\ast })/T=-\,2,-\,1,0,1,2,6.5$. The data at ${T}^{\ast }$ is marked red, the long-time result is marked in cyan. Figure created with Matplotlib v.3.1.0 (https://matplotlib.org/)^75^.

The colony can be further characterised by a measurement of the local structure. We calculated the coordination number $n$, i.e. the number of nearest neighbours, for each cell. For each simulation snapshot, we calculate a Voronoi tessellation of the cell centres $({\overrightarrow{r}}_{b}+{\overrightarrow{r}}_{f})/2$, see Fig. 6(a). To cut off the large Voronoi cells on the boundary of the colony, we intersect the Voronoi cells with circles of radius $({R}_{{\rm{\max }}}+1.5{\sigma }_{b})/2$ which represents the maximum interaction radius from the cell centre. With this corrected Voronoi tessellation, we calculate $n$ for all cells and then calculate the histogram $p(n)$, see Fig. 6(b–d). The data is shown for multiple times relative to ${T}^{\ast }$ for each ${T}_{{\rm{mot}}}$. Additionally, we show a histogram at a late stage in the simulation. The results are mostly independent of ${T}_{{\rm{mot}}}$ presented in this way.

As time progresses, cells with 6 neighbours become quickly the most prevalent, while having 3 or 4 neighbours becomes less likely. Cells with 5 neighbours lightly decline, and 7 neighboured cells become more likely. This is quite similar to the MDCK experiment^21^ and other cell types^73^. The notable difference is that our simulations overestimate configurations with 6 neighbours at long times. This is directly visible in simulation snapshots as crystallisation of the tissue. This could have many reasons such as a lack of polydispersity of cell sizes, which would hinder crystallisation, or could be caused by the strength of contact inhibition of proliferation present in the model which leads to arrest of the cells.

## Discussion

We investigated the dynamics of a colony of crawling, proliferating cells with a minimal, mechanical cell model. With a simple mechanism for contact inhibition of proliferation (CIP), we find the typical regimes of colony growth, with exponential growth at short times turning into sub-exponential growth at long times. The latter regime is characterised by the colony boundary moving outwards with a constant speed. We identify simple scaling relations for both regimes and the crossover between them.

We find that the crossover colony size ${A}^{\ast }$ between the two regimes is a marker of contact inhibition of proliferation and that the boundary speed ${v}_{B}$ in the sub-exponential growth regime expresses the strength of contact inhibition of locomotion. From Eq. (8), we see that ${A}^{\ast }$ depends on the steady state speed of the cells ${v}^{{\rm{ss}}}$ and the duration of the cell cycle $T$. The faster the cells can migrate and the longer the cell cycle is, i.e. the longer the time between division attempts, the larger the colony can become before CIP sets in. The long-time behaviour of ${v}_{B}$ only depends on the speed at which cells are able to travel, and thus primarily measures contact inhibition of locomotion (CIL), see Eq. (10). This is corroborated by the investigation of cell shape on colony dynamics. There, the crossover ${A}^{\ast }$ is unchanged by cell shape, but large-front cells are better at aligning away from the colony, and as a result, tend to be more mobile and tend to expand the colony faster at long times. It remains to be seen how close the observed faster expansion for large-front cells in the present model is to contact enhancement of locomotion^74^.

The mechanisms of CIL and CIP as exhibited by our model are idealised compared to the experimental situation. While in the experiment the bulk of cells is still mobile, our cells arrest more quickly and tend to crystallise. Also, while the mechanism of contact inhibition of locomotion – which arises from the motile force being coupled to the cell polarisation as well as from the shape of the cell itself – controls alignment of the cells away from the colony and colony growth, our cells are unable to expand the colony at their full crawling speed. We also neglected the polydispersity of cells sizes and noise.

## Methods

### Parameters

Introducing cell-cell adhesions made it necessary to modify the model’s parameters with respect to ref. ^52^. We needed to shorten the maximum extension of the cells so that the disks could not be pulled apart far enough to leave a space in-between. The changed parameters also yielded cells that more readily expand from their symmetric, circular state. We confirmed that modifying the parameters did not qualitatively change the dynamics discussed in ref. ^52^. All of the results reported here are for ${\sigma }_{f}=1.10\,{R}_{{\rm{\max }}}$ and ${\sigma }_{b}=0.87\,{R}_{{\rm{\max }}}$, for a shape ratio of ${\sigma }_{b}/{\sigma }_{f}=0.79$, except where otherwise stated. In the steady state, the cells have area ${A}_{s}=1.44\,{R}_{{\rm{\max }}}^{2}$. The division threshold is set to ${R}_{{\rm{div}}}=0.85\,{R}_{{\rm{\max }}}$. To account for the new cell-cell interactions, the energy scale is now set by ${\varepsilon }_{{\rm{core}}}$. We will discuss two cases, one with non-adhesive cells with ${\varepsilon }_{{\rm{well}}}=0$, and one with adhesive cells, with a well depth of ${\varepsilon }_{{\rm{well}}}=0.25\,{\varepsilon }_{{\rm{core}}}$. Further parameter choices are $\kappa =0.1094\,{\varepsilon }_{{\rm{core}}}/{{R}_{{\rm{\max }}}}^{2}$ and $m=0.5\,{\varepsilon }_{{\rm{core}}}/{R}_{{\rm{\max }}}^{2}$, such that $m=4.57\kappa$. This yields a steady-state distance of ${r}_{bf}^{{\rm{ss}}}=0.75{R}_{{\rm{\max }}}$ and steady-state speed ${v}^{{\rm{ss}}}={r}_{bf}^{{\rm{ss}}}m\mathrm{/(2}\zeta )=0.19\,{\varepsilon }_{{\rm{core}}}/(\zeta {R}_{{\rm{\max }}})$. All reported times are in units of $\zeta {R}_{{\rm{\max }}}^{2}/{\varepsilon }_{{\rm{core}}}$. The characteristic time scale of migration is ${\tau }_{{\rm{mig}}}={R}_{{\rm{\max }}}/{v}^{{\rm{ss}}}=5.33$. The time step of the simulations is $2.1\cdot {10}^{-3}$. The simulations are initialised with a single cell in one of the states randomly. We average our results over 10 individual simulation runs with different seeds for the pseudo-random number generator used for the calculation of the state durations and randomisation of cell orientation after a cell division.

### Cell area

The area *A* of a cell with a back particle of diameter ${\sigma }_{b}$, a front particle of diameter ${\sigma }_{f}$ and a distance ${r}_{bf}$ between the particles is given by

11 $$\begin{array}{rcl}{A}_{{\rm{cell}}} & = & {A}_{b}+{A}_{f}-{\rm{overlap}}\\  & = & \pi ({\sigma }_{b}^{2}+{\sigma }_{f}^{2})/4\\  &  & +\,\frac{1}{2}\sqrt{(\,-\,{r}_{bf}+{\sigma }_{b}+{\sigma }_{f})({r}_{bf}+{\sigma }_{b}+{\sigma }_{f})}\\  &  & \times \,\sqrt{({r}_{bf}+{\sigma }_{b}-{\sigma }_{f})({r}_{bf}-{\sigma }_{b}+{\sigma }_{f})}\end{array}$$

### Details on cell-cell interactions

**Figure 7:**
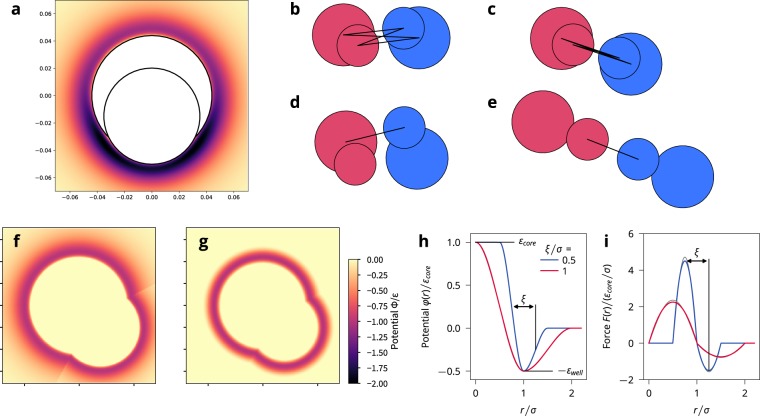
Development of a soft-core potential. (**a**) Lennard-Jones potential exerted by a cell on the disk of another cell. Note the potential minimum at the back of the smaller disk. (**b**) Illustration of the 4 possible interactions between two cells. (**c**) For two cells in contact, usually all four disks will adhere to each other, which unrealistically contracts both cells. (**d**) Illustration of allowing only the interaction between the two elements whose surfaces are closest. Analog to (**b**). (**e**) Illustration of allowing only the interaction between the two elements whose surfaces are closest. Analog to (**c**). (**f**) Potential landscape for Lennard-Jones potential around a cell in which only the closer surface is considered. (**g**) Potential landscape for the new soft potential around a cell in which only the closer surface is considered. (**h**) Potential and (**i**) force for soft-core particles with a radius of $\sigma$, energy scale ${\varepsilon }_{{\rm{core}}}$, with an attractive well of depth ${\varepsilon }_{{\rm{well}}}={\varepsilon }_{{\rm{core}}}\mathrm{/2}$, and two different well widths $\xi$. Figure created with Matplotlib v.3.1.0 (https://matplotlib.org/)^75^.

When two cells adhere to each other by the four possible interactions between the disks, potential minima can form due to superposition of the elements’ potentials at certain positions along the boundary of the cells. Those can be either behind the smaller cell element or on the sides of the cell, see Fig. 7a). This can lead to alignment of cells purely due to the potential, which we want to avoid. Additionally, cell elements can interact with other cells’ elements with which they are not in contact and even through the other cell element, since the well depth of the potential is comparable to the range of the potential, see Fig. 7b). This can cause cells which are in contact to contract each other, see Fig. 7c). In contrast, real cell interactions occur due to direct contact of cell membranes. Therefore, to accurately model cell-cell interactions we must find a way to only have surface-surface interactions as well.

We implement surface-surface interaction by letting two cells only have up to one adhesive interaction, between the two elements whose surfaces are closest, see Fig. 7(d,e). To keep the model similar to our previous paper, we still let all the cell elements repel each other.

Finally, using the Lennard-Jones potential had one additional drawback. The width of the potential well scales with the elements’ radii, which leads to a discontinuity when only the closest surface is considered adhesive and the elements have different sizes, see Fig. 7f). This was not an issue to our previous work in which the cutoff was set to make the forces purely repulsive, which makes the interaction very short ranged.

We solved these issues by switching to a soft-core potential, in which the width of the well can be set independently from the element radius, which yields a potential landscape such as the one shown in Fig. 7g). We first define a cubic helper function $t(y)$ to serve as a continuous step. The function describes a continuous and monotonous step between $t(0)=0$ and $t(\xi )=1$, in which the end points are saddle points

12 $$t(y)=\frac{{y}^{2}}{{\xi }^{3}}(3\xi -2y)$$

13 $$t{\prime} (y)=\frac{6y}{{\xi }^{3}}(\xi -y)$$

With defining $y=r-\sigma$, the force and the potential between two cell disks at separation $\overrightarrow{r}$ are given by

14 $${\overrightarrow{F}}_{sc}(\overrightarrow{r})=\{\begin{array}{cc}0, & y\le -\,\xi \\ ({\varepsilon }_{{\rm{c}}{\rm{o}}{\rm{r}}{\rm{e}}}+{\varepsilon }_{{\rm{w}}{\rm{e}}{\rm{l}}{\rm{l}}})t{\prime} (\,-\,y)\hat{r}, & -\xi \le y < 0\\ -{\varepsilon }_{{\rm{w}}{\rm{e}}{\rm{l}}{\rm{l}}}t{\prime} (y)\hat{r}, & 0\le y < \xi \\ 0, & \xi \le y\end{array}$$

and

15 $${\phi }_{sc}(r)=\{\begin{array}{cc}{\varepsilon }_{{\rm{c}}{\rm{o}}{\rm{r}}{\rm{e}}}, & y\le -\,\xi \\ ({\varepsilon }_{{\rm{c}}{\rm{o}}{\rm{r}}{\rm{e}}}+{\varepsilon }_{{\rm{w}}{\rm{e}}{\rm{l}}{\rm{l}}})t(\,-\,y)-{\varepsilon }_{{\rm{w}}{\rm{e}}{\rm{l}}{\rm{l}}}, & -\xi \le y < 0\\ {\varepsilon }_{{\rm{w}}{\rm{e}}{\rm{l}}{\rm{l}}}t(y)-{\varepsilon }_{{\rm{w}}{\rm{e}}{\rm{l}}{\rm{l}}}, & 0\le y < \xi \\ 0, & \xi \le y\end{array}$$

See also Fig. 7(h,i). The minimum of the potential is at $r=\sigma$, identifying $\sigma$ as the characteristic length scale of the potential, see Fig. 7h). ${\varepsilon }_{{\rm{well}}}$ is the energy in the minimum, i.e. $\phi (\sigma )=-\,{\varepsilon }_{{\rm{well}}}$. The distance between the two inflection points of the potential (the extremes of the force) is given by $\xi$, which is therefore a measure of the width of the potential well. We require $0\le \xi \le \sigma$ to enforce that ${\varepsilon }_{{\rm{core}}}$ is the energy for $r=0$, i.e. $\phi (0)={\varepsilon }_{{\rm{core}}}$, and that $F(0)=0$. The natural cutoff of the potential is ${r}_{{\rm{cut}}}=\sigma +\xi$.

Consider now two cells with elements $\alpha$ and $\beta$, respectively (*α, β* ∈[*b, f*]). The separation between any pair of elements $(\alpha ,\beta )$ is given by ${\overrightarrow{r}}_{\alpha \beta }={\overrightarrow{r}}_{\beta }-{\overrightarrow{r}}_{\alpha }$ with unit vector ${\hat{r}}_{\alpha \beta }={\overrightarrow{r}}_{\alpha \beta }/|{\overrightarrow{r}}_{\alpha \beta }|$. Their surface-to-surface distance is given by ${d}_{\alpha \beta }=|{\overrightarrow{r}}_{\alpha \beta }|-{\sigma }_{\alpha \beta }/2$ (with ${\sigma }_{\alpha \beta }=({\sigma }_{\alpha }+{\sigma }_{\beta })/2$) and the elements with the shortest surface-to-surface distance are denoted $(\alpha {\prime} ,\beta {\prime} )$, i.e.

16 $${d}_{\alpha {\prime} \beta {\prime} }=\mathop{{\rm{\min }}}\limits_{\alpha ,\beta }\,({d}_{\alpha \beta }).$$

Then, the (cell-cell) force ${\overrightarrow{F}}_{cc,\alpha }$ acting on cell element *α* exerted by the elements of the other cell is given by

17 $${\overrightarrow{F}}_{cc,\alpha }=\sum _{\beta }\,{\overrightarrow{F}}_{\alpha \beta }$$

18 $${\overrightarrow{F}}_{\alpha \beta }=\{\begin{array}{cc}{\overrightarrow{F}}_{sc}({\overrightarrow{r}}_{\alpha \beta }) & {\rm{i}}{\rm{f}}\,{\overrightarrow{F}}_{sc}({\overrightarrow{r}}_{\alpha \beta }){\hat{r}}_{\alpha \beta } < 0\,{\rm{o}}{\rm{r}}\,(\alpha ,\beta )=(\alpha {\rm{{\prime} }},\beta {\rm{{\prime} }})\\ 0 & {\rm{o}}{\rm{t}}{\rm{h}}{\rm{e}}{\rm{r}}{\rm{w}}{\rm{i}}{\rm{s}}{\rm{e}}\end{array}$$

using Eq. (14) for ${\overrightarrow{F}}_{sc}({\overrightarrow{r}}_{\alpha \beta })$.

With this soft-core potential, the potential well around a cell is now of constant width and of shorter range, see Fig. 7g). For all our simulations we chose a width of the potential well of $\xi =0.654\,{R}_{{\rm{\max }}}$ (=0.75*σ*_*b*_).

### Radial distribution of successful cell divisions

We calculated the radial distribution of successful cell divisions for all times, by assuming that the colony is circular, i.e. radially symmetric. We obtain

19 $${N}_{{\rm{d}}{\rm{i}}{\rm{v}}}(r,t)=\frac{1}{{A}_{{\rm{r}}{\rm{i}}{\rm{n}}{\rm{g}}}(r)}\frac{1}{\Delta t}{\int }_{t-\Delta t}^{t}{\int }_{r-\Delta r,r}\sum _{i}\delta (\overrightarrow{r}{\prime} -{\overrightarrow{R}}_{i,{\rm{n}}{\rm{e}}{\rm{w}}}(t))dr{\prime} dt$$

with the centre of mass of the colony placed at the origin, ${A}_{{\rm{ring}}}(r)=\pi [{r}^{2}-{(r-\Delta r)}^{2}]$, and ${R}_{i,{\rm{new}}}(t))$ the position of the *i*-th division event occurring at time *t*.

### Average cell orientation

We quantified the orientation of the cells in respect to the colony as follows. With $\overrightarrow{r}$ the position of the cell in respect to the centre of mass of the colony, and ${\overrightarrow{r}}_{bf}$ the cell’s extension, and $\hat{r}$ and ${\hat{r}}_{bf}$ denoting their unit vectors, we measure the orientation of cells towards or away from the colony

20 $$p(r)=\langle {\hat{r}}_{bf}\cdot \hat{r}\rangle $$

as a function of $r=|\overrightarrow{r}|$. This function yields values between −1 and 1. If cells on a ring of radius *r* are mostly pointing towards the inside of the colony, $p(r) < 0$, whereas if the cells are mostly pointing away from the colony, then $p(r) > 0$.

## Supplementary information

Supplementary Information.

Supplementary Information2.

Supplementary Information3.

## Data Availability

All data needed to evaluate the conclusions in the paper are present in the paper and the Supplementary Materials. Additional data related to this paper may be requested from the authors.
