# Supplementary material for: Control of cell colony growth by contact inhibition
Source: Sci Rep. 2020 Apr 21;10:6713. doi: 10.1038/s41598-020-62913-z (PMC7174381; doi:10.1038/s41598-020-62913-z)
Supplement: Supplementary file 1 — Supplementary Information. [file 41598_2020_62913_MOESM1_ESM.pdf]

Supplementary Material:
Control of cell colony growth by contact inhibition

Simon K. Schnyder, John J. Molina, Ryoichi Yamamoto

1 Colony growth for different cell shapes

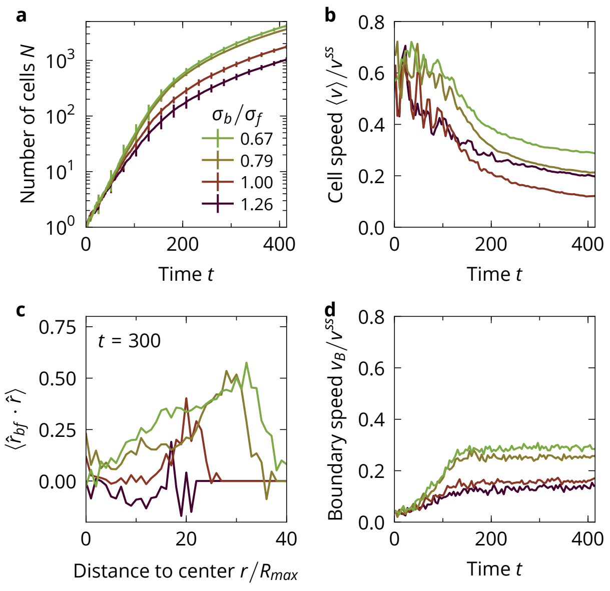

Figure S1. Colony growth for different cell shapes (non-adhesive cells). a) Number of cells as a function of time with error bars given by the standard deviation between independent simulation runs. b) Average cell speed over time. c) Average orientation of cells in respect to the direction pointing directly away from the colony center at time $t = 300$. Positive values indicate cells pointing away from the colony, negative values indicate cells pointing towards the colony. Larger values mean that cells are better aligned and/or more extended. d) Average speed of the colony boundary.

2 Movies

- Movie S2: Growing colony of non-adhesive cells with $T_{\mathrm{mot}} = 16$ and $T_{\mathrm{div}} = 3$. Cells are coloured according to the state they are in: dividing cells are blue, freshly divided migrating cells are red. Migrating cells fade to gray over a time of $t = 10$.
- Movie S3: Growing colony of adhesive cells with $T_{\mathrm{mot}} = 16$ and $T_{\mathrm{div}} = 3$ and potential-depth $\varepsilon_{\mathrm{well}} = 0.25\varepsilon_{\mathrm{core}}$. Cells are coloured according to the state they are in: dividing cells are blue, freshly divided migrating cells are red. Migrating cells fade to gray over a time of $t = 10$.
